# Utility of Weight-Bearing MRI in the Lumbar Spine: A Novel Indication

**DOI:** 10.7759/cureus.23930

**Published:** 2022-04-07

**Authors:** Taha Faruqi, William Padget, Nilesh Patel

**Affiliations:** 1 Orthopaedic Surgery, Beaumont Health, Dearborn, USA

**Keywords:** facet loading mechanics, spondylolisthesis, facet cyst, radiculopathy, weight-bearing mri

## Abstract

For decades MRI has been utilized for diagnosing spine pathology. However, like many imaging modalities utilized today, a conventional MRI is a static study. The spine is a complex, dynamic structure whose loading characteristics change with the position of the spine and the type and direction of force applied. This gives rise to dynamic pathologies that are often masked if attempted to be imaged using conventional MRIs. This is where a weight-bearing MRI (WBMRI) shines. We report the case of a 66-year-old female in whom an L3-L4 synovial facet cyst was diagnosed on a WBMRI.

## Introduction

Several pathologies of the spine are better delineated when the physiologic stress of weight-bearing is applied. For instance, MRI conducted in the upright seated position is better able to detect posterior disc herniations [1]. Other studies have corroborated these findings, suggesting that hidden nerve root compressions and hidden disc herniations can better be detected in standing MRIs [1-3]. We report a case of a patient in whom WBMRI was used to diagnose a dynamic facet cyst at the L3-L4 level.

## Case presentation

A 66-year-old female presented to our clinic with a chief complaint of low back pain for several years, which became acutely worse over the past few weeks. In addition to her chronic low back pain, she now complained of right leg radiculopathy with numbness and tingling in the right lateral thigh, knee, and leg. Her radiculopathy was exacerbated by activities such as extension and walking downstairs. No recent injury had been sustained. Past medical history was significant for sleep apnea, hypertension, chronic obstructive pulmonary disease, depression, diabetes, heart attack, chronic kidney disease, asthma, and a lower extremity deep vein thrombosis. Her back pain was eight out of ten and her right leg pain was seven out of ten. No bowel or bladder incontinence was present. She could walk for approximately five minutes before having to rest. She consumed hydrocodone/acetaminophen 10/325 mg for her chronic back pain. She underwent physical therapy two years ago and continued home exercises, although she reported minimum improvement in her symptoms from her exercises. No other treatments were undergone by the patient.

**Figure 1:**
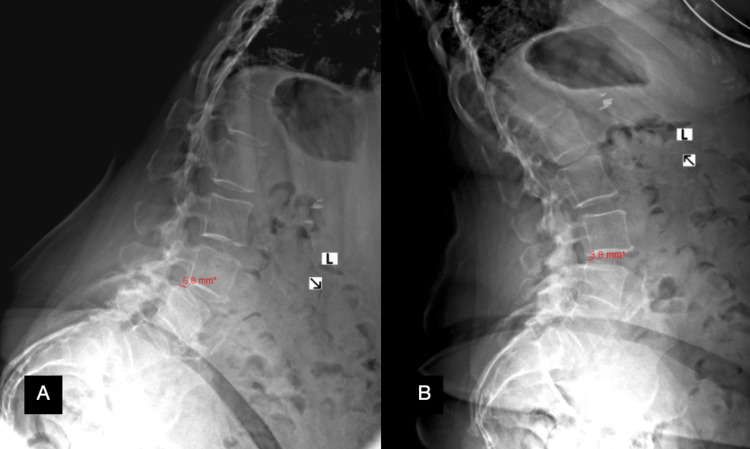
Flexion (A) and extension (B) X-ray of the lumbar spine.

**Figure 2:**
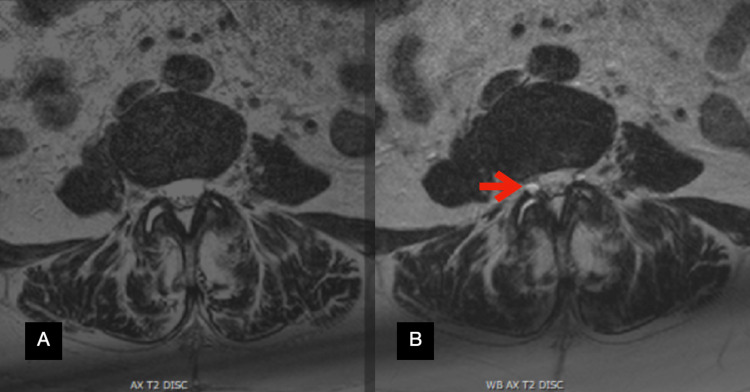
Axial T2-weighted MRIs at the L3-L4 facet joint without weight-bearing (A) and with weight-bearing (B) showing the development of a facet cyst (arrow).

Anterior-posterior (AP), lateral, flexion, and extension X-rays of the lumbosacral spine were significant for an L3-L4 grade I dynamic spondylolisthesis with 6.8 mm of listhesis on flexion (Figure 1A) and 3.8 mm of listhesis on extension (Figure 1B). A conventional MRI was obtained which did not reveal any significant pathology to explain her symptoms. Due to her dynamic spondylolisthesis and tall L3-L4 disc space, it was then opted to obtain a WBMRI. On the non-weight-bearing portion of the MRI, T2-weighted axial slices at the L3-L4 level demonstrated fluid in the facet joints, particularly in the right L3-L4 facet joint (Figure 2A). When weight bearing was simulated and the MRI was repeated, the same axial cut now demonstrated a facet cyst at the right L3-L4 facet joint (Figure 2B).

The patient was scheduled to undergo a total knee arthroplasty (TKA) within the next few weeks of her office visit and deferred any spinal surgical intervention until she recovered from her TKA. She elected to undergo epidural steroid injections in the interim. If her symptoms did not improve or if she developed any acute neurological deficits, it was decided that an L3-L4 decompression and fusion would be performed.

## Discussion

Several imaging modalities are utilized in the field of spine surgery to elucidate spinal pathology. Most imaging modalities are static in nature and hence are excellent at revealing static pathologies. Dynamic imaging is utilized to diagnose dynamic pathologies. Of these, flexion-extension X-rays of the spine are routinely used. WBMRIs, however, are not commonly employed. Even though the use of WBMRIs has been documented in the literature, to our knowledge, there have been no other reports of a patient with minimal disc degeneration and facet hypertrophy with a dynamic spondylolisthesis that has been evaluated with a WBMRI demonstrating a synovial cyst. We, therefore, propose a new indication for obtaining a WBMRI.

In our patient, after an initial conventional MRI revealed no underlying pathology explaining the patient’s symptoms, a WBMRI was performed due to tall L3-L4 disc space, a spondylolisthesis, and radiculopathy being aggravated by extension. Although symptoms worsening with extension could indicate spinal stenosis, the tall disc space hinted at a lower likelihood of intradiscal pathology and drew our attention to the posterior elements such as the ligamentum flavum and facet joints. The dynamic spondylolisthesis raised suspicion for an incompetent facet articulation. Being posterior elements of the spinal column, facets joints are offloaded with flexion and loaded with extension [4]. In fact, in their finite element analysis of the lower lumbar spine, Schmidt et al. demonstrated that while facet joints remained unloaded in flexion, facet joints experienced forces up to 50 N during extension. Further, these forces increased to a maximum of 105 N in axial rotation [5]. In their biomechanical analysis, Kuo et al. demonstrated that with an applied preload, a facet joint experiences increased forces in contralateral axial rotation [6]. This sheds some light on why it is important to obtain WBMRIs in patients presenting with postural radiculopathy, especially in the setting of an equivocal conventional MRI.

Beyond radiculopathy, WBMRIs can help to better characterize disc herniations and spondylolistheses, especially if they are dynamic. Spondylolisthesis by itself can be evaluated on flexion-extension radiographs. However, the effects of these pathologies on nearby neural elements can be underrepresented by conventional MRIs. In their study, Ferreiro Perez et al. illustrated the superiority of upright MRIs by showing that posterior disc herniations and anterolisthesis were either underestimated or missed in 58% of patients undergoing conventional recumbent MRIs [1]. Therefore, additional information can be obtained by WBMRIs that can be vital to study particularly if patients are being prepared for surgery.

Central canal stenosis is yet another aspect of spinal pathology that can be better highlighted with WBMRIs. Multiple studies assessing upright MRIs have demonstrated that a decrease in foramina, lateral recess, and central canal cross-sectional areas is caused by extension [7-8]. Schmid et al. demonstrated a central canal cross-sectional area reduction of 5.2% in normal subjects when transitioning from a supine extended to an upright extended position [9]. In the absence of facet pathology, the primary contributor of this reduction of central canal cross-sectional area is attributed to laxity of the ligamentum flavum which can increase in thickness from 3.3 mm to 4.3 mm when standing in an upright extended position from a supine position [10]. This can exacerbate symptoms of spinal stenosis in patients who already have a decreased central canal cross-sectional area owing to other pathologies such as disc herniation.

While WBMRIs impart valuable additional information, they have a few drawbacks that need to be considered prior to ordering this imaging modality. The availability of WBMRIs is not widespread. Furthermore, the cost can be prohibitive. Since multiple images with and without the weight-bearing sequence are obtained, this can, unfortunately, lead to an increased cost of the study. As an example, as compared to conventional MRI facilities, imaging conducted in upright MRI facilities in Washington State billed 2.5 times higher as per Chung et al. [11]. Furthermore, since certain WBMRIs are obtained in a standing upright position, given the long duration of image acquisition, it can be difficult for symptomatic patients to maintain an upright posture. This can result in poor non-diagnostic imaging, image artifacts, or an altogether inability to complete the study [12]. It is, therefore, imperative to correctly identify patients who would benefit from this imaging modality.

## Conclusions

Weight-bearing MRI is a modality with exciting prospects. Its ability to delineate occult spinal pathology makes it a very useful tool in diagnosing patients, especially if patients have already obtained a conventional MRI which fails to elucidate spinal pathology that correlates with their symptoms. However, obtaining WBMRIs is not feasible for every patient. We suggest obtaining a WBMRI in patients presenting with postural radiculopathy with a tall disc space and with an associated spondylolisthesis, especially in the setting of a negative conventional MRI. This case study demonstrates one of the many ways in which a WBMRI can aid in diagnosing dynamic pathology in the lumbar spine.
